# *Mycobacterium avium Complex*-Associated Hemophagocytic Lymphohistiocytosis in a Sickle Cell Patient: An Unusual Fatal Association

**DOI:** 10.1155/2013/291518

**Published:** 2013-05-16

**Authors:** Mohammed A. R. Chamsi-Pasha, M. Chadi Alraies, Abdul Hamid Alraiyes, Eric D. Hsi

**Affiliations:** ^1^Department of Internal Medicine, Cleveland Clinic, 9500 Euclid Avenue, Cleveland, OH 44195, USA; ^2^Cleveland Clinic Lerner College of Medicine of Case Western Reserve University, Department of Hospital Medicine, Cleveland Clinic, 9500 Euclid Avenue, Cleveland, OH 44195, USA; ^3^Department of Pulmonary, Critical Care and Environmental Medicine, Tulane University Health Sciences Center, 1430 Tulane Avenue, SL-9, New Orleans, LA 70112, USA; ^4^Section Head, Hematopathology, Chairman, Department of Clinical Pathology, Cleveland Clinic, 9500 Euclid Avenue Cleveland, OH 44195, USA

## Abstract

Hemophagocytic lymphohistiocytosis (HLH) is a rare hyperinflammatory syndrome, characterized clinically by fever, splenomegaly, cytopenia, and high ferritin. Infectious causes have been associated with secondary HLH, with viruses being the most common. We report a case of *Mycobacterium avium complex*-associated HLH in a sickle cell anemia patient. To the best of our knowledge, this association has never been reported in sickle cell anemia.

## 1. Introduction

HLH is a rare inflammatory disease caused by macrophage and cytotoxic T-cell activation leading to uncontrolled hemophagocytosis and cytokine production. It can be divided into primary (genetic) causes, idiopathic, and secondary causes associated with infections, hematological malignancies, and autoimmune diseases [1]. *Mycobacterium avium complex* (MAC) is a nontuberculous mycobacterium known to cause pulmonary and disseminated disease in patients with immunodeficiency. We report the first case of HLH in the setting of MAC infection in a sickle cell anemia patient, initially presenting with fever of unknown origin.

## 2. Case Presentation 

**Figure 1:**
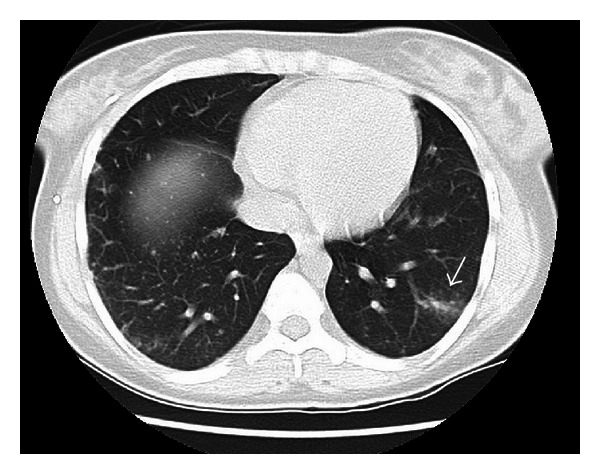
Compute tomography (CT) of chest showing wedge-shaped ground glass opacity in the left lower lobe, with surrounding centrilobular nodules (arrow). Additional 1-2 mm centrilobular nodules noted in the right lower lobe.

**Figure 2:**
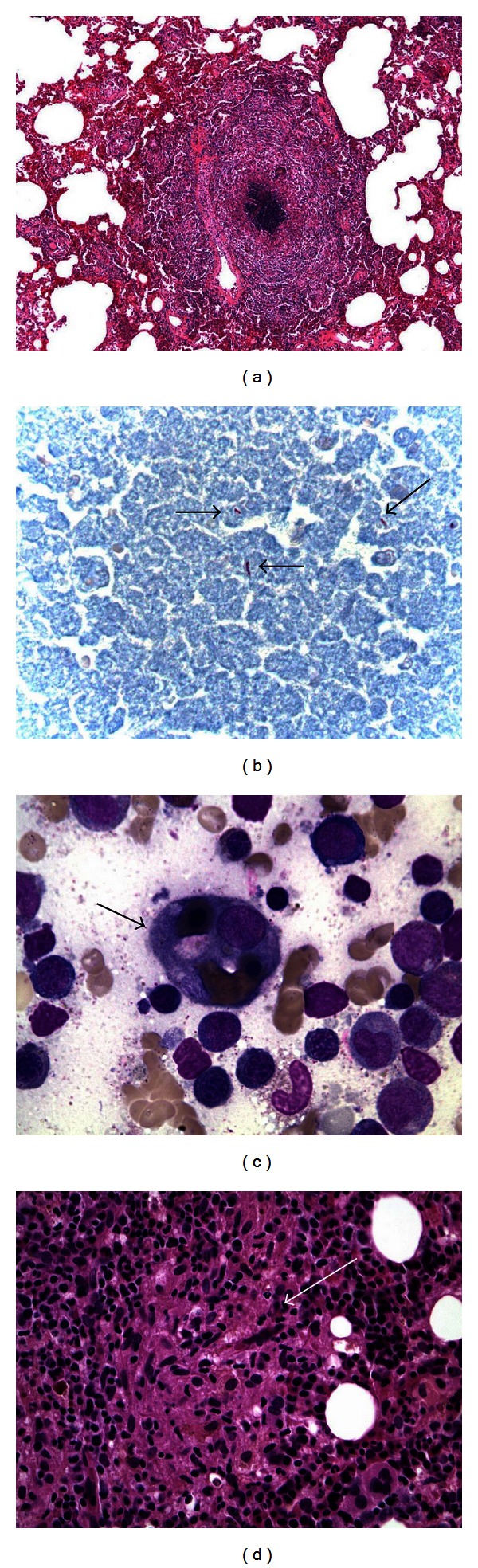
(a) Necrotizing granuloma evident on wedge-resected lung tissue. (b) Ziehl-Neelsen stain revealing acid-fast bacilli in resected lung tissue, consistent with mycobacteria (arrows). (c) Bone marrow aspirate specimen showing a hemophagocyte containing erythrocytes and pronormoblasts (arrow) (Wright stain, 1000x). (d) Core bone marrow showing a loose Granuloma (arrow) (Hematoxylin-Eosin stain, 400x).

A 22-year-old female with sickle cell anemia presented with 1-month history of fever, night sweats, and weight loss. On presentation, vital signs were stable except for fever of 102 F. Cardiopulmonary examination was normal, and there was no lymphadenopathy. The rest of physical examination was unremarkable. Laboratory studies showed leukocytosis of 17.000/uL and hemoglobin of 10.4 g/dL. Erythrocyte sedimentation rate and C-reactive protein were elevated at 44 mm/hr and 8.47 mg/dL, respectively. Patient had negative bacterial and fungal cultures, viral serologies, and tuberculin skin testing. Chest X-ray showed bilateral nodular opacities, and computed tomography (CT) revealed ground glass opacity in the left lower lobe with surrounding nodules (Figure 1). Bronchoalveolar lavage with biopsies showed granulomas with no organisms. Wedge biopsy was pursued and histopathology showed necrotizing granulomas (Figure 2(a)) with acid-fast bacilli (Figure 2(b)). Polymerase chain reaction and tissue cultures were positive for MAC, and patient was started on azithromycin, ethambutol, and rifampin. 

One month after discharge, she was readmitted with high fevers and hypoxia concerning for severe sepsis. Labs showed leukocytosis of 35.000/uL, alanine transaminase 224 U/L, aspartate transaminase 592 U/L, alkaline phosphatase 480 U/L, and cholestatic jaundice (total bilirubin 10.2 mg/dL). Severe sepsis was suspected, and patient was placed empirically on moxifloxacin, amikacin, azithromycin, and rifampin. CT of the chest showed multifocal pneumonia. Infectious workup revealed MAC in the blood with negative viral and bacterial cultures. Her course was complicated by persistent fevers, splenomegaly, and development of disseminated intravascular coagulopathy (DIC). An elevated ferritin of 38,539 ng/mL and triglyceride of 566 mg/dL were noted. Bone marrow biopsy showed histiocytes with engulfed erythrocytes and nucleated marrow elements (Figure 2(c)), along with scattered granulomas (Figure 2(d)). Bone marrow cultures were positive for MAC. A diagnosis of MAC-associated HLH was made, and patient was started on methylprednisolone and interleukin-1 receptor antagonist (Anakinra) with immediate cessation of fever spikes. However, the patient's respiratory status deteriorated and passed away soon after. 

## 3. Discussion

HLH is a rare syndrome resulting in massive activation of macrophages and cytotoxic T cells. Diagnosis is established if five out of eight criteria are met: fever; splenomegaly; cytopenia involving at least two lines; increased triglycerides and/or decreased fibrinogen; hemophagocytosis in a bone marrow, liver, or node specimen; low NK-cell activity; ferritin more than 500 ng/mL; and elevated soluble interleukin 2 receptor [2]. Our patient's ferritin value of 38,539 ng/mL strongly supports the diagnosis of HLH, and the presence of hemophagocytic cells on bone marrow biopsy is consistent with this condition. 

HLH has been associated with infections, with Epstein-Barr virus being the most common [1]. Rare cases of nontuberculous mycobacterial infections have been reported in association with HLH [3], but to date we present the first case of MAC-associated HLH in sickle cell patient.

Disseminated MAC is a common opportunistic infection in HIV-infected or immunodeficient patients but never reported in adult sickle cell anemia patients.

There is no gold standard therapeutic regimen for secondary HLH. The aim is to suppress the inflammatory response using immunosuppressive agents. However, treating the underlying disease is necessary to expedite recovery and response to therapy [1]. Agents adopted by the Histiocyte Society include combination of dexamethasone, etoposide, and intrathecal methotrexate [2]. In our case, high bilirubin levels and DIC precluded the use of etoposide and intrathecal methotrexate, respectively. The patient was started on interleukin-1 receptor antagonist, which has been shown to improve symptoms in autoinflammatory diseases [2].

This case aims to increase clinician's awareness of HLH in the setting of infectious process, as mortality rates are high and early diagnosis and treatment are mandated to improve outcomes [4].
